# Editorial: Graph Embedding Methods for Multiple-Omics Data Analysis

**DOI:** 10.3389/fgene.2021.762274

**Published:** 2021-09-20

**Authors:** Wei Lan, Qingfeng Chen, Yi-Ping Phoebe Chen, Wilson Wen Bin Goh

**Affiliations:** ^1^School of Computer Electronical and Information, Guangxi University, Nanning, China; ^2^Department of Computer Science and Information Technology, La Trobe University, Melbourne, VIC, Australia; ^3^School of Biological Sciences, Nanyang Technological University, Singapore, Singapore

**Keywords:** multi-omics data analysis, graph embedding, computational methods, data integration, disease

With the advent of advanced high throughput biotechnologies such as next-generation sequencing and single-cell sequencing, there has been an increasing growth of complex multiple-omics data sets (such as genomics, epigenome, transcriptomics, proteomics, metabolomics, etc.). These data are often heterogeneous, sparse, high dimension and high noise, which provide different levels of insightful information for disease. Integrative analysis of multiple-omics data can help the biomedical researchers to explore biological mechanisms and further assist in designing better diagnostic tools and therapies for the treatment of diseases. However, the development of effective methods for multiple-omics data analysis is very challenging as the complex characteristics of different kinds of data. Recently, machine learning methods especially graph embedding have shown powerful capability in analyzing multiple-omics data. Particularly, they are capable to represent data as low dimensional vectors while the data features are preserved.

To provide a platform bridging graph embedding method and multiple-omics data analysis, we organized we organized a Research Topic on “*Graph Embedding Methods for Multiple-Omics Data Analysis*.” This Research Topic presents 19 articles. We expect that these articles promote more advanced studies for multiple-omics data analysis.

Peng L. et al. identified potential antiviral drugs against SARS-CoV-2 by using regularized least squared classifier and bipartite local model. Ninety six virus-drug associations between 11 types of viruses similar to SARS-CoV-2 and 78 small molecular drugs were extracted in this study.

Hou et al. proposed a method to capture potential functions in a microbial co-occurrence network. It integrated topological structures of microbial co-occurrence networks with k-mer compositions of operational taxonomy unit sequences and embedded them into a lower-dimensional continuous latent space.

Pan et al. presented an embedding-based method for predicting the subcellular localization of proteins. The functional and network embeddings from GO terms and protein–protein network were combined as novel representations of protein locations for the construction of the final classification model.

Gu et al. proposed a method incorporating feature engineering and feature selection algorithms to explore the common controlling genes and corresponding pathways among eight different organs' fibrosis. These results were helpful for understanding the molecular mechanisms of fibrosis diseases and finding new therapeutic indications of existing drugs.

Zhang developed a feature selection algorithm for gene expression data classification by using approximate conditional entropy based on fuzzy information granule. The experimental results on large-scale gene datasets show that this algorithm not only greatly reduces the dimension of the gene datasets, but also is superior to the state-of-the-art algorithms in classification accuracy.

Yuan and Yang proposed a deep learning method to identify circRNA-RBP interactions by using hybrid double embeddings for representing RNA sequences and a cross-branch attention neural network for classification. The experimental results on benchmark datasets show that their method outperforms the mainstream deep learning-based methods on not only prediction accuracy but also computational efficiency.

He et al. adopted multiple kernel learning (MKL) to integrate somatic mutation to currently molecular data including gene expression, copy number variation (CNV), methylation, and protein expression data for the prediction of breast cancer survival. In addition, the maximum relevance minimum redundancy (mRMR) feature selection method was utilized to select features that present high relevance to survival and low redundancy among themselves for each type of data.

Su et al. designed a multi-level model to improve both the quality and speed of large-scale PPIs prediction. The results showed that their model is promising for large-scale PPI prediction in both accuracy and efficiency, which is beneficial to other large-scale biomedical molecules interactions detection.

Barbiero et al. tested the digital twin model on two simulated clinical case studies combining information at organ, tissue, and cellular level. The results show their approach is able to detect inflammatory cytokines which are known to have effects on blood pressure and have previously been associated with SARS-CoV-2 infection (e.g., CXCR6, XCL1, and others).

Zhang et al. developed a computational method based on the Light Gradient Boosting Machine (LightGBM) to predict potential metabolite–disease associations. It extracted the features from statistical measures, graph theoretical measures. Three case studies confirmed that this method has obvious superiority in predicting metabolite–disease pairs and represents a powerful bioinformatics tool.

Zhao et al. proposed a supervised gene selection method based on permutation and random forest classification. The experimental result on 10 datasets show that the gene selection performance of their method is better than other gene selection methods.

Wang J. et al. presented a computational drug repositioning approach to discover potential drug-disease associations. The experimental results demonstrate that their approach outperforms recent state-of-the-art prediction models. In addition, the case studies further confirm the predictive ability of the proposed method.

Wang, Dai, et al. introduced a pan-cancer classification method to identify a set of genes that can differentiate all tumor types accurately. Extensive experimental results on the public RNA-seq data sets with 33 different tumor types show that this method outperforms the other state-of-the-art classification methods.

Wang, Cao, et al. proposed a computational method to predict and identify the m6A sites on mRNA by utilizing sequence-derived and graph embedding features. The comparison results show that the proposed method achieved the best performance compared with other predictors on four public datasets across three species.

Wang Z. et al. explored the gene expression changes and its potential effects mediated by U11 snRNA in bladder cancer cell. This study show that U11 may be involved in the regulation of gene expression in bladder cancer cells, which may provide a potentially new biomarker for clinical diagnosis and treatment of bladder cancer.

Feng et al. integrated transcriptomic, lipidomic, and metabolomic analyses to identify the differential lipids and metabolites between basal and luminal muscle invasive bladder cancer (MIBC) subtypes. The results suggest that free fatty acids (FFA) and sulfatides (SL), which are closely associated with immune and stromal cell types, have strong capacities to distinguish basal and luminal subtypes of MIBC tumors. Moreover, the results also show that the ratios of glycerophosphocholine (GCP)/imidazoles and nucleosides/imidazoles can accurately identify tumors of basal and luminal MIBC subtypes.

Peng X. et al. presented a method to construct methylation haplotypes for homologous chromosomes in CpG dense regions. The proposed method not only can be applied to methylation analysis, but also can provide a clear explanation for the methylation difference at the resolution of methylation haplotypes.

Liu and Zhang developed a computational model for the detection of copy number variation detection (CNV) of different lengths from whole genome sequencing data. It used a clustering algorithm to divide the read depth segment profile, and assigned an abnormal score to each read depth segment. The experimental results show that the performance of proposed model is better than those of several existing methods.

Zheng and Wu proposed a method for predicting drug-target interactions based on heterogeneous network integration and cascade deep forest. The results show that their model outperforms the previously reported methods on the benchmark datasets.

## Author Contributions

The article was written by WL and QC. Y-PC and WG have provided guidance to the manuscript preparation and have also reviewed and edited the paper. All authors have approved the final version of the editorial.

## Funding

This work was partially supported by the National Natural Science Foundation of China (Nos. 62072124, 61963004, and 61972185), the Natural Science Foundation of Guangxi (Nos. 2021GXNSFAA075041 and 2018GXNSFBA281193), the Science and Technology Base and Talent Special Project of Guangxi (No. AD20159044).

## Conflict of Interest

The authors declare that the research was conducted in the absence of any commercial or financial relationships that could be construed as a potential conflict of interest.

## Publisher's Note

All claims expressed in this article are solely those of the authors and do not necessarily represent those of their affiliated organizations, or those of the publisher, the editors and the reviewers. Any product that may be evaluated in this article, or claim that may be made by its manufacturer, is not guaranteed or endorsed by the publisher.

